# Principal Component Analysis Applied to In-School Inertial Measurement Unit-Derived Data During Physical Activity: A Systematic Review Highlighting Children’s Behavioral Patterns

**DOI:** 10.3390/s26082542

**Published:** 2026-04-20

**Authors:** Daniel González-Devesa, Markel Rico-González, Daniel Rojas-Valverde, Carlos D. Gómez-Carmona

**Affiliations:** 1 Research Group on Physical Activity, Education, and Health (GIAFES), Catholic University of Ávila, 05005 Ávila, Spain; 2 Department of Didactics of Music, Plastic and Body Expression, University of the Basque Country (EHU), 48940 Leioa, Spain; 3 Centro de Investigación, Desarrollo e Innovación en Salud y Deporte (CIDISAD), Escuela Ciencias del Movimiento Humano y Calidad de Vida (CIEMHCAVI), Universidad Nacional, Heredia 40101, Costa Rica; drojasv@una.cr; 4 Research Group in Training, Physical Activity and Sports Performance (ENFYRED), Department of Music, Plastic and Body Expression, University of Zaragoza, 44003 Teruel, Spain; carlosdavid.gomez@unizar.es

**Keywords:** PCA, statistics, technology, physical activity, children

## Abstract

**(1) Background:** Given the large amount of data extracted from information technologies, principal component analysis (PCA) allows the identification of the most important variables to assess physical activity (PA). The aim of this systematic review is to highlight which variables, extracted through PCA (as a data reduction technique), provide the most information about preschool and school children’s PA-related behavioral patterns during school hours. **(2) Methods:** The search was conducted in PubMed, SCOPUS, Web of Science, and ProQuest Central according to the PRISMA guidelines and the guidelines for performing systematic reviews in sports sciences. **(3) Results:** From 403 studies initially identified, seven were fully reviewed, and their outcome measures were extracted and analyzed. An analysis of these seven studies (n = 8927) revealed that volume-related components explained the majority of the variance (62.8–69.0%) in PA behaviors, while intensity components contributed less (14.4–14.8%). However, confidence intervals and heterogeneity statistics (I^2^) were not reported in the original studies, limiting quantitative synthesis. **(4) Conclusions:** This systematic review demonstrates that PCA effectively identifies multidimensional patterns in children’s PA and motor development, with volume-related dimensions consistently dominating the variance structure across diverse populations and settings.

## 1. Introduction

Engaging in physical activity during childhood is consistently associated with benefits across physical [1], mental [2], and social development [3]. Despite these well-documented benefits, levels of physical inactivity and sedentary behavior remain high [4]. For this reason, the World Health Organization recommends that children aged 5–17 accumulate an average of ≥60 min/day of moderate-to-vigorous physical activity (MVPA) [5]. However, the available evidence suggests that only a small proportion of children meet the physical activity recommendations in this regard [6]. These patterns underscore the need for scalable, evidence-based strategies to promote daily physical activity in childhood.

Establishing healthy habits during school years is critical, as behaviors adopted in childhood tend to persist into adulthood [7]. Given that children spend a considerable proportion of their daily time in school, this setting represents a strategic environment for promoting health literacy and increasing opportunities for physical activity [8]. Numerous studies have examined school-based physical activity interventions across various domains, including recess [9], physical education classes [10], active breaks [11], and active commuting [12], and many of these have used objective methods, such as pedometers [13] and accelerometers [14], to quantify physical activity, thereby generating large datasets.

Designing effective, evidence-based strategies requires a precise understanding of influencing variables and the contexts in which school-aged children accumulate physical activity. These movement behaviors are complex and multidimensional, shaped by interrelated factors such as intensity, duration, bout structure, temporal patterns, environmental characteristics, and child-level characteristics [15,16].

Principal component analysis (PCA) is a statistical dimensionality reduction method that transforms a set of highly correlated variables into a smaller number of orthogonal components that explain most of the system’s variance [17]. Each component is a weighted linear combination of the original variables, ordered so that the first explains the greatest variance and subsequent components explain progressively less [18]. By summarizing redundant information into a few interpretable dimensions, PCA facilitates pattern detection and comparison across individuals or contexts [17,18]. From this viewpoint, it could be hypothesized that children’s activity patterns can be summarized through PCA by two main components, “total activity volume” and “activity intensity”, rather than requiring an analysis of dozens of individual time periods.

In the study of children’s and adolescents’ movement behaviors, PCA can help to identify latent activity-related patterns by condensing complex information from objective movement data, as well as school- and commuting-related behaviors, into a smaller set of interpretable components [19,20,21]. In this way, PCA can be used to derive latent profiles of physical activity that are not apparent in univariate or bivariate analyses [17]. This approach has already been applied in physical exercise settings—for example, to identify session-based and weekly microcycle profiles in training and competition in youth players [22,23]; to reduce and prioritize indicators for training monitoring and talent identification in soccer, basketball, and rugby across studies including mixed age groups [24]; to characterize intensity distributions and effort variability in endurance sports in adults [25]; and to summarize health-related fitness test batteries in children and adolescents to generate percentiles and multivariate profiles [26].

Despite the growing use of PCA in the study of physical activity, to the best of our knowledge, no systematic review has yet synthesized its specific application to data collected during school hours. Therefore, the aim of the systematic review is to highlight which variables, extracted through PCA (as a data reduction technique), provide the most information about preschool and school children’s PA-related behavioral patterns during school hours. The resulting information may help to identify latent patterns, behavioral profiles, and key contexts, providing valuable evidence for the design and evaluation of school-based interventions and for decision-making by teachers and educational policymakers.

## 2. Materials and Methods

### 2.1. Study Design

The PRISMA (Preferred Reporting Items for Systematic Reviews and Meta-Analyses) standards [27] and guidelines for conducting systematic reviews in sports sciences [28] were followed to conduct this systematic review (see Supplementary Materials). The protocol of this systematic review was registered retrospectively in PROSPERO (CRD420251270900).

### 2.2. Information Sources

To find publications published before 1 October 2025, a thorough search of four major databases—PubMed, ProQuest, SCOPUS, and Web of Science—was carried out. In addition, the reference lists of all included studies were screened to identify further eligible articles. Corresponding authors were contacted when the full text was not available.

### 2.3. Search Strategy

The question was explicitly stated using the PICO (Patient, Problem, or Population–Intervention or Exposure–Comparison, Control, or Comparator–Outcome[s]) design. The search was restricted to scientific articles/journals and the English language whenever feasible (see exclusion criterion number 6). Journal names and the authors of manuscripts were not hidden. The search approach was applied to the aforementioned databases. After downloading each article, they were examined for eligibility using the inclusion–exclusion criteria one at a time. An article was downloaded and added to the review, and its data were extracted, if it satisfied all inclusion requirements. An article was removed with a detailed explanation if it did not satisfy all inclusion requirements. The following search terms were used to search articles’ titles and abstracts (see Table 1):

*(Preschool OR kindergarten OR “primary education” OR “elementary education” OR school OR schoolchildren) AND (“PCA”) AND (“physical education” OR “physical activity”).*

### 2.4. Eligibility Criteria

One author (M.R.-G.) retrieved the information (title, authors, date, and database) from the papers and entered it into an Excel spreadsheet (Microsoft Corporation, Redmond, WA, USA) to eliminate duplicates. To identify publications that satisfied all inclusion requirements, the remaining articles were screened by two authors (D.G.-D. and C.D.G.-C.) (Table 1). During this process, if these two authors did not agree on whether an article should be included/excluded, a third author (D.R.-V.) made the decision. All authors involved in this process had extensive experience in conducting systematic reviews.

### 2.5. Data Extraction

An Excel spreadsheet was used to perform data extraction in compliance with the Cochrane Consumers and Communication Review Group’s data extraction template. All of the chosen studies were evaluated according to the inclusion and exclusion criteria using the spreadsheet. All extracted data were stored in a single, centralized spreadsheet that could be reviewed simultaneously by the authors. For each included record, information was extracted regarding the sample, intervention, tests/instruments, variables, main findings, and authors’ conclusions.

Data extraction was performed independently by two reviewers (C.D.G.-C. and D.G.-D.). A third reviewer (M.R.-G.) was available for consultation when needed. Both reviewers independently extracted data from all 7 included studies. Disagreements were resolved through discussion between the two primary reviewers. Inter-rater agreement for key data elements (sample characteristics, PCA methodology, variance explained, and main outcomes) was substantial (Cohen’s κ = 0.89, 95% CI: 0.82–0.96).

### 2.6. Quality of Studies

The Methodological Index for Non-Randomized Studies (MINORS) was used to assess methodological quality [29]. Eight core elements constitute the MINORS scale, which is expanded to twelve items for comparative research. Nine appropriate measures (maximum score: 18 points) were used to evaluate methodological quality in the current evaluation; three items were considered not applicable (NA). The methodological quality of each item is represented by a score between 0 and 2 (2 = high, 1 = moderate, and 0 = low).

## 3. Results

### 3.1. Identification and Selection of Studies

**Figure 1:**
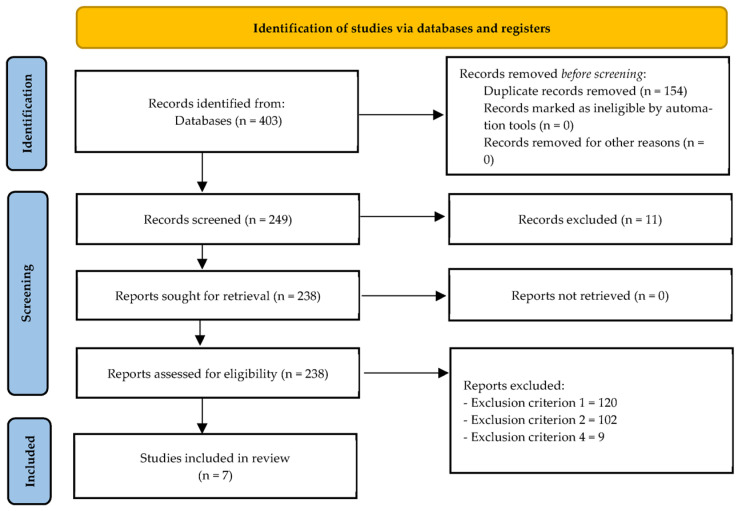
Flow diagram of the study.

After retrieving 403 original articles from PubMed (n = 57), ProQuest (n = 28), SCOPUS (n = 222), and Web of Science (n = 96), 154 duplicates were eliminated, leaving 249 unique items. Eleven articles were eliminated due to failing to meet inclusion criterion six after title and abstract screening. After reviewing the entire texts of the remaining 238 articles, 120, 102, and 9 articles were excluded according to exclusion criteria one, two, and four, respectively. As a result, seven publications [30,31,32,33,34,35,36] met all inclusion requirements and were included in the final qualitative synthesis (see Figure 1).

### 3.2. Quality Assessment

The methodological quality assessment of the seven included studies, evaluated using the MINORS criteria, revealed generally high standards across the body of evidence (Table 2). Among the three comparative studies, two achieved excellent methodological quality (Aadland et al. [30]: 21/24; Culková and Dušková [34]: 19/24), while one demonstrated acceptable quality (Parvinpour et al. [36]: 16/24). Regarding the four non-comparative studies, three attained excellent quality ratings (Geraci and Farcomeni [35]: 14/16; Belton et al. [31]: 13/16; Rocha et al. [32]: 13/16), and one showed acceptable quality (Clark et al. [33]: 12/16).

Common methodological strengths across studies included clearly stated aims, appropriate endpoint selection, and adequate follow-up periods. However, notable limitations were identified consistently across all included studies, particularly the absence of prospective sample size calculations (item 8) and, in some cases, inadequate consecutive patient inclusion (item 2) or loss to follow-up exceeding 5% (item 7). Despite these limitations, the overall methodological quality of the evidence base was deemed sufficient to support the synthesis of the findings, with five out of seven studies (71.4%) achieving excellent quality ratings according to the respective MINORS thresholds.

### 3.3. Study Characteristics

Seven studies met the inclusion criteria for this review, comprising a total of 8927 participants. The studies were conducted across diverse geographical regions, including Norway [30], Ireland [31], the United Kingdom [32,33,35], the Czech Republic [34], and Iran [36]. Sample sizes varied considerably, ranging from 17 participants in the study of Parvinpour et al. [36] to 5682 participants in the study of Geraci and Farcomeni [35].

The majority of studies employed cross-sectional designs [30,31,33,35], with one longitudinal comparative study examining transitions between educational settings [34], one experimental comparative study [36], and one cross-sectional study with activity mapping [32]. Participant ages ranged from preschool children (mean age 4.3 years) [7] to primary school children (mean age 10.5 years) [32], with most studies focusing on early-to-middle childhood periods. The sex distribution was relatively balanced across studies, with male participants comprising between 50 and 58.5% of the samples in most investigations.

Specific populations were targeted in several studies, including children from areas of low socioeconomic status [31], children with developmental delays [36], and children transitioning from kindergarten to primary school [34]. Weight status varied across samples, with most participants classified as normal-weight, although overweight and obese children were represented in multiple studies [31,33].

### 3.4. Measurement Methods and Variables

#### 3.4.1. Measurement Devices and Specifications

Physical activity assessment methods varied across studies, with accelerometry being the predominant approach (6/7 studies). Complete device specifications are presented in Table 3.

ActiGraph accelerometers (ActiGraph LLC, Pensacola, FL, USA) were employed in five studies [30,31,33,35], utilizing models GT1M, GT3X, or GT3X+ with sampling frequencies of 30–100 Hz. Different studies reported technical specifications and data collection procedures for these devices [37,38,39], although none were dedicated validity studies. Devices were waist- or hip-mounted (midaxillary line) using elastic belts as per manufacturer guidelines.

Custom MEMS accelerometers with ADXL345 sensors (Analog Devices, Norwood, MA, USA) were used in two studies [32,33] for specialized movement analysis, featuring a range of ±16 g, 40 Hz sampling, and ankle-mounted placement. Barnes et al. (2017) employed this sensor to quantify children’s movement mechanics and activity patterns [40].

Digital pedometers (inSPORTline Strippy, Prague, Czech Republic) were employed in one study [34]. Device placement involved the ankle or wrist, as per manufacturer instructions; however, specific placement for individual children was not documented, representing a methodological limitation affecting data validity, as the mounting location has been shown to affect activity recognition and step count accuracy [41]. Finally, 3D motion capture (MyoMotion, Noraxon USA Inc., Scottsdale, AZ, USA) with nine IMUs was utilized for kinematic analysis [36].

#### 3.4.2. Data Collection Protocols

Wear time criteria varied by study design. Free-living monitoring ranged from 7 to 14 days [30,35], with minimum requirements of ≥8–10 h/day and ≥3–4 days/week. School-based monitoring included specific hours (7:30 AM–2:00 PM) [34] or recess periods (40–50 min) [32]. Laboratory assessment involved controlled catching trials [36].

Non-wear time was defined as ≥20–60 consecutive minutes of zero counts [30,31,33,35] or not reported [32,34,36]. Data were collected during waking hours, excluding water activities. Epoch lengths ranged from 1-s [30,33] to 5-s intervals [31], with some studies analyzing continuous raw data [32].

#### 3.4.3. Statistical Analysis Approaches

PCA was the primary statistical method across all studies, with varying implementations. Standard PCA with rotation (varimax) was used in three studies [31,33,36]. Multivariate pattern analysis combined PCA with partial least squares regression [30] or dynamic time warping [32]. Probabilistic PCA with quantile regression addressed missing data mechanisms [35].

Component retention criteria included the Kaiser criterion (eigenvalue >1.0) [31,33], scree plots [35], or variance thresholds (>90%) [36] or were not reported [30,32,34]. Standardization (zero-centering, unit variance) was explicitly documented in three studies [33,35,36]. Additional techniques included hierarchical clustering [32], machine learning for activity classification [32,33], cross-validation [34], and quantile regression [35].

#### 3.4.4. Physical Activity Variables and Outcomes

Temporal patterns included time of day (morning, after school, evening) [31] and weekday versus weekend comparisons [31,35]. Intensity distributions used 12–17 count-per-minute bins depending on age [30], with intensity gradients calculated as log-transformed slopes [30].

Movement quality metrics included spectral purity [33], kinematic synergies (14 degrees of freedom) [38], and motion event parameters (Δt, Δd) from dynamic time warping [32]. Volume measures encompassed daily steps [34], acceleration counts [35], and time in sedentary/light/MVPA categories [31,35].

Motor competence outcomes were assessed using the TGMD-2/3 [30,36] and MABC-2 [33]. Health outcomes included composite cardiometabolic scores [30]. Covariates included the school environment [34], family lifestyle [34], child temperament [34], anthropometrics [33,35], and temporal factors (season, sex) [35].

### 3.5. Main Outcomes

#### 3.5.1. PCA Methodology and Variance Structure

PCA revealed distinct patterns in children’s physical activity behaviors. Components retained varied from two [30,33,36] to eight [35], with the total variance explained ranging from 51.2% [31] to approximately 98% [34]. Volume-related components consistently explained the largest proportion of variance: 62.8% (ASK, n = 841, 10 year olds) and 69.0% (PRESPAS, n = 1081, preschoolers) [30]. Intensity components contributed 14.4% and 14.8%, respectively, with combined solutions explaining 77.3–83.8% of the total variance [30]. Two-component solutions also distinguished movement factors (PC1: 27.0% variance, α = 0.93; loadings 0.718–0.882) from anthropometric factors (PC2: 19.2% variance, α = 0.91; loadings 0.777–0.950) in 65 preschoolers [33]. MABC-2 classification showed five children in the red zone (7.7%), 10 in amber (15.4%), and 50 in green (76.9%).

Temporal patterns demonstrated different structures for weekdays versus weekends. In 213 children, weekday patterns required three components (51.2% variance, KMO = 0.708), capturing morning, school transitions, and after-school periods, while weekend patterns required four components (60.4% variance, KMO = 0.725) [31]. Probabilistic PCA in 5682 seven year olds [35] identified eight components explaining 87.1% of the variance, with PC1 = 40.7% (predominant weekday vs. weekend behavior) and PC2 = 9.5% (weekend patterns). A kinematic analysis in 17 children with developmental delays [36] revealed two components explaining 91.2% of the variance (PC1 = 72.3%, PC2 = 18.9%), representing coordination synergies.

A model performance comparison in 841 10 year olds and 1081 preschoolers [30] showed the following: multivariate pattern analysis explained R^2^ = 20.5% (cardiometabolic health) and R^2^ = 7.4% (motor skills); standard PCA explained R^2^ = 17.4% and R^2^ = 6.5%; intensity gradients explained R^2^ = 14.0% and R^2^ = 6.1%. Machine learning with PCA in 24 children [32] showed that PC1 correlated with movement intensity (r = 0.54) and PC2 reflected temporal phasing, and hierarchical clustering identified 30 activity clusters.

#### 3.5.2. Physical Activity Patterns and Determinants

A time-of-day analysis in 213 children [31] revealed quartile differences (Q4-Q1): weekday after-school durations were 48.72 min (males) and 32.48 min (females); weekend evening durations were 32.52 min (males) and 10.84 min (females). A mixed ANOVA revealed time effects (males: η^2^p = 0.713, *p* < 0.001; females: η^2^p = 0.748, *p* < 0.001) and quartile × time interactions (males: η^2^p = 0.273, *p* < 0.001; females: η^2^p = 0.202, *p* = 0.04). Sex differences were shown, with males having higher MVPA across daily (*p* = 0.002), weekday (*p* = 0.01), and weekend (*p* = 0.02) periods. Guideline adherence was 65.5% (males) versus 46.4% (females) (χ^2^ = 7.88, *p* < 0.001, φ = −0.19), with an overall value of 56.7%. Boys were more active across all quantiles among 5682 children [35], with stronger effects in upper quantiles.

An analysis of the kindergarten-to-primary-school transition in 43 children [34] showed a 22.7% step reduction from 35,554 to 27,470 per week (Wilcoxon W = 474, *p* < 0.001, r = 0.487). Regression with four PCA components (~98% variance) identified child temperament (β = 6801.08, SE = 1571.56, t = 4.328, *p* < 0.001) as significant; family lifestyle (β = 241.25, *p* = 0.706) and athletes in the family (β = 3592.21, *p* = 0.161) were not. Spearman correlations with steps were as follows: passive school conditions r = 0.77 (*p* < 0.001), active conditions r = 0.56 (*p* < 0.001), temperament r = 0.47 (*p* < 0.01), family factors r = 0.13 (ns).

A seasonal analysis in 5682 children [35] showed autumn PA decreases versus summer, with larger effects in upper quantiles. A missing data analysis showed weekday total counts β = −4.682 (SE = 0.162) and MVPA time β = 2.794 (SE = 0.105), as well as weekend total counts β = 4.239 (SE = 0.090) and MVPA time β = −1.759 (SE = 0.054). Anthropometric measurements showed minimal effects on PC scores.

#### 3.5.3. Health, Motor, and Developmental Outcomes

An analysis of cardiometabolic health in 841 10 year olds [30] (composite from six variables: blood pressure, lipids, glucose, insulin, waist–height ratio, fitness) showed the following: PCVolume β = −0.27 (*p* < 0.001), PCIntensity β = 0.31 (*p* < 0.001), intensity gradient β = −0.38 (*p* < 0.001). Model R^2^ was as follows: intensity gradient = 14.0%, PCA = 17.4%, multivariate pattern = 20.5%. Activity at 7000–7999 cpm showed the highest selectivity ratio. An analysis of motor skills in 1081 preschoolers [30] (TGMD-3 locomotor sum scores) showed the following: PCVolume β = 0.25 (*p* < 0.001), PCIntensity not significant, intensity gradient β = 0.25 (*p* < 0.001). Model R^2^ was as follows: intensity gradient = 6.1%, PCA = 6.5%, multivariate pattern = 7.4%. Activity at 10,000–10,999 cpm showed the strongest association.

A motor skill intervention in 17 children with developmental delays [36] showed TGMD-2 catching changes: intervention 2.5 ± 1.88 versus control 0.4 ± 0.51 (t = 2.91, *p* < 0.05). A chi-squared test showed that 90% (11/12) in the intervention versus 20% (1/5) of controls progressed to upper levels (χ^2^ = 6.08, *p* < 0.05). Post-intervention kinematic analysis showed different synergy patterns in the intervention group, with the reorganization of 14 joint degrees of freedom.

## 4. Discussion

This systematic review synthesized current evidence regarding the application of PCA to objectively measured in-school physical activity in children. The seven included studies demonstrated that PCA is a critical tool for data reduction in reducing complex, high-dimensional movement datasets into a few components that reveal meaningful behavioral structures. The synthesis of seven studies (n = 8927) revealed that PCA consistently identified a dominant component related to the total volume of physical activity, explaining 62.8–69.0% of the variance, while secondary components related to intensity accounted for 14.4–14.8% of the total variability. These findings are consistent with those of other studies using PCA as a clustering strategy [42].

The findings indicate that the amount of accumulated movement (activity) remains the primary differentiator of children’s physical behavior, as seen in the two studies providing these specific variance breakdowns (n = 1922 total: ASK n = 841, PRESPAS n = 1081) [32]. However, this pattern requires confirmation in larger, more diverse samples across different age groups, settings, and populations. The intensity of movement, while contributing significantly to overall patterns, accounted for a smaller proportion of variance across the included studies. This is supported by evidence suggesting that schools should consider high-intensity physical activity and sports in their curricula, due to the positive association between high-intensity physical activity and physical fitness, mainly in children and adolescents [43]. Notably, in one study examining multivariate approaches [30], these methods demonstrated high utility when explaining cardiometabolic and skill-related health outcomes when compared to traditional cut-point MVPA measures. For example, intensity bins of between 9000 and 10,999 counts per minute showed the strongest associations with cardiometabolic health and locomotor skill development [30].

These results support the argument that surveillance systems and organizations should transition away from single-summary metrics of MVPA and adopt cut-point-free data representations capable of capturing the full complexity of children’s movement behavior [44]. This shift has potential implications for school health programming, as it may allow for the more accurate identification of responsive behavioral targets for intervention. The generalizability of current latent PA behavior classifications remains limited by geographical and demographic gaps across the available evidence. The majority of included studies originated from European high-income settings, with a single study conducted in Iran. In this sense, low- and middle-income regions, particularly in Africa, Latin America, and Southeast Asia, were not represented. Most samples consisted predominantly of White children who were enrolled in well-resourced educational systems. These contextual patterns restrict broader applicability and suggest that behavioral structures identified via PCA may differ significantly in environments where access to safe outdoor spaces, recreation facilities, and structured school PA opportunities is limited [45]. Considering that the issue of inadequate physical activity among children and adolescents spans significant social, cultural, political, legal, and economic factors, it cannot be effectively addressed while focusing on a single group or isolated strategy [46]. Efforts aimed at expanding this research to diverse settings are critical to ensure that behavioral models inform equitable and contextually relevant health promotion strategies worldwide.

Evidence-based studies consistently revealed poor to low adherence to international physical activity recommendations in children [47,48]. In this sense, in the included studies, PCA allowed a deeper understanding of when activity was accumulated, revealing that, even among those reaching the recommended daily totals, most moderate-to-vigorous activity occurred outside core instructional hours, e.g., in the study by Belton et al. [31]. Weekday after-school periods and weekend evening windows appeared to be highly influential behavioral segments that produced the largest differences between the most and least active children [31]. These findings suggest practical implications: despite representing the most universal health promotion setting available to children, schools are currently underutilized with respect to structured and spontaneous PA opportunities [49]. Considering the above, more real-world studies are needed when approaching this problem [50].

Sex-based differences in PA patterns were observed consistently, as in other studies. Boys accumulated more daily PA across all temporal segments and demonstrated greater engagement in vigorous activities in the analyzed investigations. Guideline compliance (≥60 min MVPA per day) was significantly higher in males (65.5%) compared to females (46.4%) (χ^2^ = 7.88, *p* < 0.001, φ = −0.19) [31]. Moreover, these disparities were particularly evident among the most active children, suggesting that girls are largely absent from the highest-intensity segments of play. These sex differences in PA during weekdays and school hours suggest the need for targeted interventions encouraging engagement in physical activity, particularly among girls [51]. This discrepancy may exacerbate the development of a negative feedback loop in which reduced skill proficiency and movement confidence suppress future engagement in high-intensity play, contributing to long-term inequities in health trajectories [52]. Interventions designed within school systems must therefore address the socio-behavioral barriers faced by girls, incorporating accessible play structures, skill-building opportunities, and socio-emotional support to promote confidence and social acceptance of active participation [53].

Environmental and seasonal conditions were also shown to substantially influence children’s PA in the reviewed studies. One longitudinal study (n = 43) found that the transition from kindergarten to primary school produced a 22.7% reduction in step counts during school hours [34], driven predominantly by school environmental characteristics such as access to active recess, equipment availability, and teacher-supported activity. However, this finding requires replication in larger, more diverse samples before drawing definitive conclusions about policy implications. Additionally, seasonal analyses in a larger cohort (n = 5682) indicated that PA declined markedly during autumn relative to summer [35], with the largest reductions observed among children who were typically the most active. Collectively, these results demonstrate that children’s movement behavior is highly sensitive to environmental affordances and climatic constraints. For school health promotion, this highlights the potential importance of ensuring weather-resilient outdoor play spaces, policy support for movement-integrated learning, and flexible scheduling to maintain opportunities for vigorous PA throughout the year [54,55]. Moreover, adults and systems should encourage children’s outdoor play, considering that research suggests that the natural environment offers diverse and challenging play and exploration opportunities that allow significant development in children [56]. 

A consistent theme emerging from the reviewed evidence is the fundamental role of intensity and movement quality in promoting child health and development. Beyond metabolic adaptations, high-intensity bursts appear to provide essential neuromotor stimuli for bone loading, muscle strengthening, and the acquisition of fundamental motor skills. This supports the principle that motor competence emerges not solely from increasing the volume of movement but through appropriately challenging dynamic interactions that encourage multi-joint synergy formation. Therefore, promoting vigorous play, diverse affordances, and coordination-rich tasks within school environments may be crucial in optimizing developmental outcomes.

Taken together, the findings of this review emphasize that PCA reveals significant disparities across populations, intensities, and environments that are largely obscured by traditional PA assessment methods. Based on these findings, health promotion and education professionals should consider translating these insights into targeted action. Schools should increase access to structured and unstructured vigorous activities during the school day and ensure that recess and physical education programs explicitly foster motor skill development. Policies must prioritize the protection of PA during educational transitions, particularly from early childhood education into primary school, where one study (n = 43) detected substantial declines, although these require confirmation in larger samples [34]. Sex-responsive strategies are essential to promote equitable access to vigorous movement among girls, given the consistent sex differences observed across multiple studies [31,35]. Based on the finding that high-intensity activity contributes uniquely to cardiometabolic health and motor skill outcomes [30], future intervention studies should examine the optimal timing, duration, and frequency of intensity bursts during school hours. The climate-adaptive design of physical environments must ensure sustained PA opportunities throughout the year, as seasonal variations significantly affect PA levels, particularly among the most active children [35].

## 5. Limitations and Future Research Directions

Although behavioral pattern analysis provides valuable insights into how multiple movement behaviors interact to influence children’s health, several limitations must be acknowledged. A critical limitation is the substantial methodological heterogeneity in PCA techniques, which directly affects results’ comparability and interpretation. The included studies utilized different PCA approaches—standard PCA with various rotation methods (varimax, none, or not reported), probabilistic PCA, and multivariate pattern analysis—each with distinct mathematical assumptions. Furthermore, studies applied different criteria for component retention (eigenvalue >1, scree plot, variance explained), selected different input variables (raw counts vs. intensity bins vs. temporal segments), and employed varying preprocessing steps (standardization documented in only 3/7 studies). This heterogeneity means that components labeled similarly (e.g., “volume component”) may not represent identical constructs, limiting direct synthesis. The variance explained by components is particularly sensitive to the number and types of input variables, making cross-study comparisons inherently challenging.

Heterogeneity in measurement protocols further influenced consistency. Accelerometer devices (ActiGraph models, custom MEMS, pedometers), wear time criteria (≥8 to ≥10 h/day), and epoch lengths (one second versus five seconds) varied substantially across studies, affecting temporal pattern representation and movement variability capture. Additionally, the predominant use of cross-sectional designs (6/7 studies) restricts causal inference regarding relationships between movement patterns and health outcomes. The limited number of eligible studies (n = 7) and their geographical concentration (predominantly European high-income settings) highlight the need for broader and more standardized applications of PCA in diverse school-based populations. This limitation suggests that future studies should share a common framework. In fact, future research works could establish a research framework to apply in studies with children at school. However, it should be noted that, in contrast to other fields, where several methodological studies have led to guidelines for applying PCA in sports sciences [42], the publication of a research framework for children in school should be preceded by studies across different lines of research. Moreover, the use of technological devices such as inertial measurement units within schools is scarce, at least in comparison with other fields. Research teams are encouraged to address this with the aim of establishing a common protocol.

Additional limitations include the following: (1) incomplete device specifications in some studies (validation references not reported for all devices), preventing a full reproducibility assessment; (2) the absence of test–retest reliability data for PA measurements in most studies; (3) confidence intervals and heterogeneity statistics (I^2^, Q-test) not reported in any study, precluding a meta-analysis; (4) retrospective rather than prospective protocol registration; and (5) none of the included studies reported prospective power calculations, raising concerns about sample size adequacy for detecting meaningful effects in PCA analyses. In addition, (6) we highlight the limited assessment of publication bias due to the small sample size (n = 7). In fact, the number of publications is the greatest limitation, limiting the ability to fully extrapolate, contextually analyze, understand, and generalize. Hence, the interpretation of the results extracted in this systematic review should be performed with caution, pending future research studies that corroborate them. In particular, from the 8927 participants that participated in the included studies, 5682 were included in one study, while the remaining children represented the other six studies.

Future research would benefit from consensus guidelines on PCA implementation, including standardized variable selection, component retention criteria, and reporting standards to enhance comparability. Specifically, future studies should consider the following: extend PCA applications to underrepresented populations in low- and middle-income countries, particularly in Africa, Latin America, and Southeast Asia; conduct longitudinal investigations to establish causal relationships between PCA-derived movement patterns and health outcomes; investigate whether behavioral profiles can predict intervention responsiveness to enable personalized physical activity prescriptions; and explore advanced dimensionality reduction techniques (e.g., tensor decomposition, deep learning approaches) to capture temporal dynamics and individual trajectories in physical activity behavior.

## 6. Conclusions and Practical Applications

Despite the limited number of eligible studies (n = 7) and the methodological heterogeneity, as acknowledged in the Limitations section, this systematic review provides valuable insights into how PCA can be used to effectively identify multidimensional patterns in children’s physical activity and motor development. An analysis of seven studies (n = 8927) revealed that volume-related components consistently explained the majority of the variance (62.8–69.0%) in PA behaviors, while intensity components contributed less (14.4–14.8%), across different age groups and settings. Multivariate pattern analysis outperformed traditional summary measures, explaining 20.5% of the variance in cardiometabolic outcomes versus conventional MVPA metrics. Key findings from the synthesized evidence include the following: after-school weekday and weekend evening periods showed the greatest activity disparities (33–49 min) between the most and least active children; kindergarten-to-primary-school transitions produced significant PA decreases (22.7%, *p* < 0.01), which were driven by school environmental factors rather than family lifestyles; and novel accelerometer-derived metrics (spectral purity, machine learning classification) demonstrated strong validity (α = 0.93) as automated motor competence proxies. Persistent sex disparities (guideline compliance in boys was 65.5% vs. 46.4% in girls), seasonal variations, and quantile-specific associations underscore the potential value of targeted, context-sensitive interventions addressing specific subpopulation needs.

Interventions should prioritize school environmental modifications, including enhanced equipment availability, organized active recess, and integrated movement opportunities throughout the day, as these factors primarily determine children’s activity levels during educational transitions. Teachers and physical education instructors should implement specific strategies including the following: (1) incorporating 30-s high-intensity activity bursts every 10 min during lessons to accumulate vigorous PA; (2) organizing skill-building stations during PE that emphasize participation and effort over competitive performance to engage less active children; (3) integrating 2–3-min movement breaks between academic classes; and (4) establishing after-school supervised activity clubs during the critical 2–9 PM window, when activity disparities are greatest (33–49 min between active and inactive children). Coaches should (1) design practice sessions where children spend >50% of time in moderate-to-vigorous activity rather than waiting or listening; (2) use small-sided games (3v3 or 4v4) to maximize individual participation and movement; (3) implement progressive skill challenges that build motor competence and confidence in less skilled children; and (4) ensure equitable equipment access and space utilization through structured rotation systems.

Both teachers and coaches must create psychologically safe environments through praise for effort and improvement rather than outcomes, offering activity choices that appeal to diverse interests (particularly for girls), and organizing mixed-ability groupings that prevent the exclusion of less active children. They should target after-school weekday hours (2–9 PM) and weekend evenings, which show the greatest disparities between active and inactive children. They should also implement differentiated strategies aimed at engaging girls in vigorous activities and maintaining PA during autumn/winter months. Moreover, they should utilize automated accelerometer-based movement quality assessments for resource-efficient, large-scale motor competence monitoring. For children with developmental delays, it is recommended to implement 8-week constraint-based interventions (2 × 30-min sessions weekly) emphasizing functional coordination patterns over isolated skill instruction. Policy initiatives must address kindergarten-to-primary-school transitions through active learning integration, outdoor lessons, and movement breaks to counteract the significant PA declines during this critical developmental period.

## Figures and Tables

**Table 1:** Inclusion and exclusion criteria for study selection.

No.	Item	Inclusion Criteria	Exclusion Criteria	Search Terms
1	Population	Preschool or schoolchildren attending education centers	Children aged more than 12 years old (from middle school or high school) Children out of school (ambulatory children, children receiving medical treatment)	Preschool OR kindergarten OR “primary education” OR “elementary education” OR school OR schoolchildren
2	Intervention/Exposure	Children attending educational centers when data were registered during at least part of the data registration period (24 h data registers are included) During school hours, physical activity data are registered using objective tools with a specified brand/manufacturer and model (e.g., accelerometer, pedometer, inertial measurement units, motion capture systems)	Studies that only include data extracted outside of school Children not participating in physical activity sessions Studies that use PCA to establish associations but not for extracting movement patterns Physical activity data extracted from questionnaires Studies that use consumer-grade devices (e.g., fitness trackers, smartwatches) without research applications Staff experiences Studies based on surveys	“physical education” OR “physical activity”
3	Comparison	-	-	
4	Outcome(s)	Outcomes extracted from PCA or factor analysis applied to movement or physical activity data for dimensionality reduction	Outcomes not extracted from PCA or equivalent multivariate analysis Datasets that include non-physical activity data or mixed physical activity data with others (nutritional, sleep, etc.) Cluster analysis and discriminant analysis as primary analyses (classification methods, not dimensionality reduction).	“PCA”
5	Study Design	-	-	
6	Other Criteria	Peer-reviewed, original, full-text original articles	Non-peer-reviewed Non-original articles Conference proceedings Dissertations Books Study protocols Reviews	

Note: PCA = principal component analysis.

**Table 2:** Methodological assessment of the included studies.

Reference	1	2	3	4	5	6	7	8	9	10	11	12	Score
Aadland et al. [30]	2	2	2	2	2	2	1	0	2	2	2	2	21/24
Belton et al. [31]	2	1	2	2	2	2	2	0	NA	NA	NA	NA	13/16
Clark et al. [33]	2	1	2	2	2	2	1	0	NA	NA	NA	NA	12/16
Culková and Dusková [34]	2	1	2	2	1	2	2	0	2	2	1	2	19/24
Geraci and Farcomeni [35]	2	2	2	2	2	2	2	0	NA	NA	NA	NA	14/16
Parvinpour et al. [36]	2	1	2	2	1	1	1	0	2	1	1	2	16/24
Rocha et al. [32]	2	1	2	2	2	2	2	0	NA	NA	NA	NA	13/16

**Note:** NA = not applicable. **The MINORS checklist** (2 = high quality; 1= medium quality; 0 = low quality): clearly defined objective (item 1); inclusion of patients consecutively (item 2); information collected retrospectively (item 3); assessments adjusted to the objective (item 4); evaluations carried out in a neutral way (item 5); follow-up phase consistent with the objective (item 6); dropout rate during follow-up less than 5% (item 7); prospective estimation of sample size (item 8); adequate control group (item 9); simultaneous groups (item 10); homogeneous starting groups (item 11); and appropriate statistical analysis (item 12).

**Table 3:** Characteristics of included studies.

Reference	Sample	Measurement Methods	Physical Activity Assessment	PCA	Main Outcomes/Variables	Results (*p* < 0.05)	Conclusions
Aadland et al. [30] **Country:** Norway **Design:** Comparative cross-sectional study	**Participants (n):** 841 (ASK) + 1081 (PRESPAS) **Final sample (n):** 841 (ASK) + 1081 (PRESPAS) **Sample size calculation:** NR **Sex:** ASK: 50% boys, 50% girls; PRESPAS: 52% boys, 48% girls **Age, years (mean; SD):** ASK: 10.2 (0.3); PRESPAS: 4.7 (0.9)	**Model:** ActiGraph GT3X+ accelerometer **Manufacturer:** ActiGraph LLC, Pensacola, FL, USA **Range:** NR **Validation:** NR **Sampling frequency and epoch:** 30 Hz, 1 s **Placement:** Waist-worn (above right hip)	**Duration:** 7 days (ASK), 14 days (PRESPAS) **Wear time criteria:** ≥8 h/day, ≥4 days/week **Non-wear time:** Zero count >20 min (PRESPAS), >60 min (ASK) **Setting:** Free-living, waking hours except water activities, 06:00–23:59	**Objective:** Reduce PA intensity spectrum to simple metrics for association analysis **Rotation:** NR (orthogonal constraint specified) **Components retained:** 2 (criteria NR) **Standardization:** Log-transformed; further standardization NR	***Independent variables*** PA intensity bins (ASK, 12 bins, 0–99 to >10,000 cpm; PRESPAS, 17 BINS (0–99 TO >15,000 cpm) Intensity gradient (slope of ln time vs. ln intensity) Principal components (PCVolume, PCIntensity) Overall PA (cpm) ***Dependent variables*** Composite cardiometabolic health score (ASK dataset) Locomotor skills score (PRESPAS dataset)	***PCA*** *PCVolume:* ASK = 62.8%, PRESPAS = 69.0% *PCIntensity:* ASK = 14.4%, PRESPAS = 14.8% *Total:* ASK = 77.3%, PRESPAS = 83.8% ***Associations with Cardiometabolic health (ASK)*** Intensity gradient: β = −0.38, *p* < 0.001, R^2^ = 14.0% PCVolume: β = −0.27, *p* < 0.001 PCIntensity: β = 0.31, *p* < 0.001 PCA model: R^2^ = 17.4% Multivariate pattern (PLS): R^2^ = 20.5%, strongest at 7000–7999 cpm ***Associations with Locomotor Skills (PRESPAS)*** Intensity gradient: β = 0.25, *p* < 0.001, R^2^ = 6.1% PCVolume: β = 0.25, *p* < 0.001 PCA model: R^2^ = 6.5% Multivariate pattern (PLS): R^2^ = 7.4%, strongest at 10,000–10,999 cpm	Volume-related PA components explained majority of variance (63–69%), while intensity explained less (14–15%) All cut-point-free approaches outperformed traditional MVPA summary measures Multivariate pattern analysis provided best model fit for both health and motor outcomes Specific intensity bins showed stronger associations than summary measures
Belton et al. [31] **Country:** Ireland (Northern Ireland and Republic of Ireland) **Design:** Cross-sectional observational study	**Participants (n):** 408 recruited **Final sample (n):** 213 (met wear time criteria) **Sample size calculation:** NR **Sex:** 54.5% male **Age, years (mean; SD):** 8.7 (0.5) **BMI (mean; SD):** 19.1 (4.5) kg/m^2^ **Population:** Primary school children from low socioeconomic status areas	**Models:** ActiGraph GT1M, GT3X, or GT3X+ **Manufacturer:** ActiGraph LLC, Pensacola, FL, USA **Range:** NR **Validation:** NR **Sampling frequency and epoch:** NR, 5-s **Placement:** Hip-mounted (midaxillary line)	**Duration:** 8 days **Wear time criteria:** ≥10 h/day, ≥3 weekdays + 1 weekend day **Non-wear time:** ≥20 min of zero counts **Setting:** Free-living, y-axis data, first day omitted	**Objective:** Identify temporal PA patterns from hourly MVPA time blocks **Rotation:** Varimax **Components retained:** 3 (weekday), 4 (weekend); Kaiser criterion (eigenvalue ≥1.0); KMO test applied; loadings ≥0.3 considered important **Standardization:** NR	***Independent variables*** Time blocks (PCA-derived): ○ Weekdays (morning, 8–9 AM; school transitions, 9–10 AM, 1–2 PM; after school, 2–9 PM) ○ Weekends (early morning, midday, afternoon, evening) • Sex-specific MVPA quartile classification ***Dependent variables*** MVPA (min/day): daily, weekday, weekend	***PCA*** *Weekday:* 3 PCs (51.2% variance, KMO = 0.708) *Weekend:* 4 PCs (60.4% variance, KMO = 0.725) ***Guideline achievement and sex differences*** *MVPA adherence:* 56.7% met guidelines; males 65.5% vs. females 46.4%, χ^2^ = 7.88, *p* < 0.001, φ = −0.19 *Sex differences:* males > females in daily (*p* = 0.002), weekday (*p* = 0.01), weekend MVPA (*p* = 0.02) ***ANOVA (Quartile × Time)*** *Males:* Time effect *p* < 0.001, η^2^p = 0.713; interaction *p* < 0.001, η^2^p = 0.273 *Females:* Time effect *p* < 0.001, η^2^p = 0.748; interaction *p* = 0.04, η^2^p = 0.202 ***Quartile differences (Q4-Q1)*** *Males:* weekday after-school 48.72 min, weekend evening 32.52 min *Females:* weekday after-school 32.48 min, weekend midday 10.84 min	After-school (weekdays) and evening (weekends) are critical “activity-rich” periods showing greatest disparities between most and least active children Interventions should target these specific time windows, especially for girls and least active children from low-SES backgrounds PCA successfully identified distinct temporal PA patterns
Clark et al. [33] **Country:** United Kingdom **Design:** Cross-sectional observational study	**Participants (n):** 65 **Sample size calculation:** NR **Sex:** 38 boys (58.5%), 27 girls **Age, years (mean; SD):** 4.3 (0.7) **Height (m; SD):** 1.04 (0.05) **Weight (kg; SD):** 17.8 (3.2) **BMI (kg/m^2^; SD):** 16.2 (1.9) **Weight status:** Underweight n = 3, normal n = 40, overweight n = 13, obese n = 9	***Device 1*** **Model:** Custom MEMS with ADXL345 sensor **Manufacturer:** Analog Devices, Norwood, MA, USA **Range:** ±16 g **Validation:** NR **Sampling frequency and epoch:** 40 Hz, continuous **Placement:** Ankle (lateral malleolar), Velcro strap ***Device 2*** **Model:** ActiGraph GT3X+ **Manufacturer:** ActiGraph LLC, Pensacola, FL, USA **Range:** NR **Validation:** NR **Sampling frequency and epoch:** 100 Hz, 1 s epochs **Placement:** Waist (above right hip), elastic belt	**Duration:** Free-play recess, 100 ± 3 min/day **Wear time criteria:** NR (single session) **Non-wear time:** ≥20 min of zero counts **Setting:** Outdoor preschool recess	**Objective:** Investigate movement quality, PA, and motor competence; feature extraction with dimensionality reduction **Rotation:** Varimax with Kaiser normalization **Components retained:** 5; Kaiser criterion (eigenvalue >1.0); loadings >0.3 **Standardization:** Yes	***Variables for PCA*** Spectral purity (frequency-based movement quality) MABC-2 traffic light scores (red/amber/green zones) Fine motor percentile Gross motor percentile Anthropometric measures: weight, BMI, BMI percentile, body fat percentage ***PCA output*** PC1: Movement component PC2: Anthropometric component	***PCA (5 PC, 74.9% variance)*** **PC1 (Movement)** 27.0% variance, α = 0.93 Loadings: spectral purity (0.866), traffic light (red zone n = 5, amber zone n = 10, green zone n = 50; 0.882), fine motor (0.718), gross motor (0.790) **PC2 (Anthropometric)** 19.2% variance, α = 0.91 Loadings: BMI (0.950), BMI% (0.932), body fat% (0.897), weight (0.777) **PC3 (Age/Height)** 11.6% variance, α = 0.81 Loadings: height (0.897), age (0.789)	Spectral purity (automated accelerometric measure) clustered with traditional motor competence assessments (MABC-2), demonstrating that frequency-based movement quality metrics can serve as automated, time-saving proxies for motor competence in preschool children Clear separation between movement and anthropometric components supports use of novel sensor-based assessment
Culková and Dusková [34] **Country:** Czech Republic **Design:** Longitudinal comparative study (before–after transition)	**Participants (n):** 52 initially **Final sample (n):** 43 (21 girls, 22 boys) **Sample size calculation:** NR **Age:** Kindergarten (May 2022) → primary school grade 1 (October 2022) **Settings:** 3 kindergartens, 3 primary schools (Olomouc region) **Schools selected:** Different passive/active PA conditions	**Model:** inSPORTline Strippy digital pedometers **Manufacturer**: inSPORTline, Prague, Czech Republic **Range:** NR **Validation:** NR **Sampling frequency and epoch:** NR **Placement:** Ankle or wrist	**Duration:** 5 weekdays per phase **Wear time criteria:** School time only (7:30 AM–2:00 PM) **Non-wear time:** NR **Setting:** School only	**Objective:** Address multicollinearity; PCs used as predictors in regression for step count **Rotation:** NR **Components retained:** NR **Standardization:** NR	***Independent variables*** Gender, age School type (kindergarten vs. primary school) School environment ○ Passive conditions (facilities, equipment size, availability) ○ Active conditions (outdoor lessons, learning in motion, active breaks) • Family factors ○ Active transportation ○ Seasonal sports ○ Competitive athletes, ○ Child temperament ***Dependent variables*** Number of steps per 5-day school week	***PCA*** 4 components, ~98% total variance ***Primary outcome*** Steps decreased 22.7% from kindergarten (35,554) to primary school (27,470) Wilcoxon test: W = 474, *p* < 0.001 Effect size: r = 0.487 ***Regression predictor*** Child temperament: β = 6801.08, SE = 1571.56, t = 4.328, *p* < 0.001 ***Spearman correlations with steps*** Passive school conditions: r = 0.77, *p* < 0.001 Active school conditions: r = 0.56, *p* < 0.001 Temperament: r = 0.47, *p* < 0.01	Significant PA decrease occurs during kindergarten-to-primary-school transition, primarily driven by school environmental factors rather than family lifestyle School environments and structured activities crucially impact children’s PA Urgent need for policy interventions including improved sports equipment availability, active recess organization, and integration of PA into school day structure
Geraci and Farcomeni [35] **Country:** United Kingdom **Design:** Cross-sectional cohort study (Millennium Cohort Study)	**Participants (n):** 5682 **Sample size calculation:** NR **Population:** Singleton children, white ethnic background **Age:** 7 years **Source:** MCS longitudinal study, PA monitoring 2008–2009	**Model:** ActiGraph GT1M **Manufacturer**: ActiGraph LLC, Pensacola, FL, USA **Validation:** NR **Sampling frequency and epoch:** NR **Placement:** Waist	**Duration:** 7 days **Wear time criteria:** >10 h/day, >2 valid days **Non-wear time:** >20 min zero counts **Setting:** Free-living, waking hours except aquatic activities; mailed devices	**Objective:** Extract PA patterns accounting for non-ignorable missing data; repeated daily measurements as variables **Rotation:** NR (PPCA—orthogonal by construction) **Components retained:** 8 discussed (explaining >90% variance); scree plot presented **Standardization:** Yes	***Variables for PCA (42 variables: 7 outcomes × 7 days)*** Total acceleration counts Total steps Proportion sedentary behavior Proportion light PA Proportion MVPA Duration MVPA bouts Frequency MVPA bouts ***8 PCAs*** PC1: Predominant behavior (weekday vs. weekend) PC2–PC3: Weekend patterns PC4–PC7: Day-specific patterns PC8: Sedentariness ***Covariates analyzed*** Sex, season (spring/summer/autumn/winter) Anthropometry (height, weight, waist circumference, body fat %)	***PCA*** PC1 = 40.7%; PC2 = 9.5%; PC3 = 7.6%; PC4 = 6.9%; PC5 = 6.5%; PC6 = 6.3%; PC7 = 5.6%; PC8 = 4% Total (8 PCs) = 87.1% ***Missing data mechanism (Model ii, AIC = 471,290.8)*** Weekday: Total counts β = −4.682 (SE = 0.162), MVPA time β = 2.794 (SE = 0.105) Weekend: Total counts β = 4.239 (SE = 0.090), MVPA time β = −1.759 (SE = 0.054) ***Sex effects*** Boys more active than girls (*p* < 0.05), stronger effects in upper quantiles (most active children) ***Seasonal effects*** Marked PA decrease in autumn vs. summer, greatest effect on most active children (upper tail)	Children exhibit distinct sedentary/active behaviors with clear weekday–weekend patterns identifiable through PC scores Different activity groups can be characterized by few PC scores rather than multiple PA variables Strong sex and seasonal associations exist, with effects varying by quantile (activity level) Interventions should target autumn/winter periods and weekdays, especially for girls, and promote vigorous activities
Parvinpour et al. [36] **Country:** Iran **Design:** Experimental comparative study (control vs. constraint)	**Participants (n):** 17 **Sample size calculation:** NR **Population:** Children with developmental delays **Sex:** 7 boys, 10 girls **Age, years (mean; SD):** 5.58 (0.52) **Height (m; SD):** 1.15 (0.06) **Groups:** Control group vs. constraint manipulation group	**Model:** MyoMotion 3D motion capture (9 IMUs) **Manufacturer:** Noraxon, USA **Range:** NR **Validation:** NR **Sampling frequency and epoch:** NR **Placement:** 9 IMUs (lower/upper back, head, bilateral arms/forearms/hands), Velcro straps	**Duration:** 10 successful trials (pre/post), 5 trials (transfer); ~12 attempts per 10 successes **Wear time criteria:** NR **Non-wear time criteria:** NR **Setting:** Laboratory catching task; soft ball (16 cm), underhand throw, 2 m distance	**Objective:** Quantify kinematic synergies (14 DoFs) as functional units; examine intervention effects on coordination **Rotation:** Varimax **Components retained:** 2 main synergies; variance >90% + correlation >0.50 criteria **Standardization:** Yes (zero mean, unit variance)	***Independent variables*** Group (constraint manipulation vs. control) Intervention (8-week task constraints vs. typical PE) Test phase (pre, post) ***Dependent variables*** TGMD-2 catching scores Developmental sequence levels (arm, hand, body) 14 joint degrees of freedom (DoFs): wrist, elbow, shoulder ***PCA output (kinematic synergies)*** PC1 and PC2 joint motions	***PCA*** PC1 = 72.3%, PC2 = 18.9% Total = 91.2% (pre-test) ***TGMD-2 catching score change*** Intervention: 2.5 ± 1.88; control: 0.4 ± 0.51 t = 2.91, *p* < 0.05 **Developmental progression:** χ^2^ = 6.08, *p* < 0.05 Intervention: 90% progressed to upper levels vs. control with 20% **Synergy characteristics** Bimanual, multiaxial, multi-segmental coordination Post-intervention: Different kinematic synergies emerged in intervention group	Task constraints effectively manipulate fundamental motor skill development in children with developmental delays Challenge of multi-joint coordination dimensionality is negotiated through synergy formation maximizing spatial–temporal accuracy Constraint manipulation promotes motor skill acquisition through reorganization of coordination patterns and emergent synergies from body segments
Rocha et al. [32] **Country:** United Kingdom **Design:** Cross-sectional observational study with activity mapping	**Participants (n):** 24 children **Final sample:** 118 motion records (5 days) **Sample size calculation:** NR **Sex:** 18 boys, 6 girls **Age (mean; SD):** 10.5 (0.6) years **Height (m; SD):** 1.44 (0.09) **Weight (kg; SD):** 39.6 (9.5) **BMI (kg/m^2^; SD):** 18.8 (3.1) **Weight status:** Normal n = 16, overweight n = 5, obese n = 2, underweight n = 1 **School years:** 12 in year 5, 12 in year 6	**Model:** Custom MEMS with ADXL345 sensor **Manufacturer:** Analog Devices, Norwood, MA, USA **Range:** ±16 g **Validation reference:** NR **Sampling frequency and epoch:** 40 Hz, continuous **Placement and attachment:** Ankle (lateral malleolar), Velcro strap	**Duration:** 40 min analyzed (42–50 min recorded, standardized to first 40 min) **Wear time criteria:** 5 consecutive school days **Non-wear time criteria:** NR **Activities/setting:** School recess free play	**Objective:** Profile activity patterns; identify children with similar activity; clustering **Rotation:** NR **Components retained:** 2 (for visualization) **Standardization:** NR DTW for motion event comparison; dendrogram clustering	***Independent variables*** Motion event parameters: ○ Δt: Fractional time change between events ○ Δd: Acceleration magnitude of events • Motion traces over 40 min recess ***PCA output*** PC1: Movement intensity over time PC2: Temporal phasing ***Additional variables*** DTW distance from reference motif Cluster membership Anthropometry (height, weight, BMI)	***PCA*** PC1: Integrated movement intensity over 40 min (Pearson r = 0.54 with mean acceleration) PC2: Temporal phasing of activity ***Motion event parameters*** Δt (time change), Δd (acceleration magnitude) Event criteria: 1.1 s duration, ≥1.5 g peak, 40 samples min distance 80 metrics extracted per child (Δd in 2 min sliding window over 40 min) 2D PCA plot: clear differentiation of diverse activity profiles Regions identified: low (L), medium (M), high (H) intensity profiles ***Clustering*** 30 clusters identified (WPGMA method, Euclidean distance) Paired children in PCA plot showed highly correlated acceleration profiles Dendrogram confirmed hierarchical relationships (e.g., cluster 17)	Accelerometer-based multivariate analysis provides valid automated methodology for mapping children’s activities during play Pattern recognition and machine learning successfully classify activity types, offering objective, time-saving alternative to traditional observer assessment Method applicable for large-scale PA surveillance and research in pediatric populations

**Note:** AIC, Akaike information criterion; ASK, Active Smarter Kids; BMI, body mass index; cpm, counts per minute; DoF, degrees of freedom; DTW, dynamic time warping; IMU, inertial measurement unit; KMO, Kaiser–Meyer–Olkin (measure of sampling adequacy); MABC-2, Movement Assessment Battery for Children-2; MCS, Millennium Cohort Study; MEMS, micro-electro-mechanical system; MVPA, moderate-to-vigorous physical activity; NR, not reported; PA, physical activity; PC, principal component; PCA, principal component analysis; PE, physical education; PLS, partial least squares; PPCA, probabilistic principal component analysis; PRESPAS, Preschool Physical Activity Study; R^2^, coefficient of determination; SD, standard deviation; SES, socioeconomic status; TGMD-2, Test of Gross Motor Development-2; WPGMA, weighted pair group method with arithmetic mean; β, standardized regression coefficient; η^2^p, partial eta squared; χ^2^, chi-squared statistic.

## Data Availability

No new data were created or analyzed in this study. Data sharing is not applicable to this article.

## Supplementary Materials

The following supporting information can be downloaded at: https://www.mdpi.com/article/10.3390/s26082542/s1, Table S1: PRISMA 2020 Checklist.
